# The effects of two short-chain perfluoroalkyl carboxylic acids (PFCAs) on northern leopard frog (*Rana pipiens*) tadpole development

**DOI:** 10.1007/s10646-024-02737-z

**Published:** 2024-02-05

**Authors:** Jillian Rohonczy, Stacey A. Robinson, Mark R. Forbes, Amila O. De Silva, Cassandra Brinovcar, Adrienne J. Bartlett, Ève A. M. Gilroy

**Affiliations:** 1 Department of Biology, Carleton University, Ottawa, ON K1S 5B6 Canada; 2 Wildlife and Landscape Science Directorate, Environment and Climate Change, Ottawa, ON K1A 0H3 Canada; 3 Water Science and Technology Directorate, Environment and Climate Change Canada, Burlington, ON L7S 1A1 Canada

**Keywords:** Amphibian, PFAS, Ecotoxicology, Chronic toxicity, Sublethal effect

## Abstract

Short-chain perfluoroalkyl carboxylic acids (PFCAs) have been detected in the environment globally. The presence and persistence of these compounds in the environment may lead to chronic wildlife exposure. We used northern leopard frog (*Rana pipiens*) tadpoles to investigate the chronic toxicity and the bioconcentration of two short-chain PFCAs, perfluorobutanoic acid (PFBA) and perfluorohexanoic acid (PFHxA). We exposed Gosner stage 25 tadpoles to PFBA and PFHxA (as individual chemicals) at nominal concentrations of 0.1, 1, 10, 100, and 1000 µg/L for 43–46 days. Tadpoles exposed to 0.1 to 100 µg/L of PFBA and PFHxA had significantly higher mean snout-to-vent lengths, mean masses, and scaled mass indexes than control tadpoles. These results indicate that exposure to short-chain PFCAs influences tadpole growth. Further investigation into the mechanism(s) causing the observed changes in tadpole growth is warranted. We observed a significantly higher proportion of males in the PFBA 1 µg/L treatment group, however further histological analyses are required to confirm visual sex identification before making concrete conclusions on the effects of PFCAs on amphibian sex ratios. PFBA concentrations in tissues were higher than PFHxA concentrations; a pattern that contrasts with previously published studies using fish, suggesting potential differences between taxa in PFBA and PFHxA bioconcentration. Bioconcentration factors were <10 L/kg wet weight, indicating low bioconcentration potential in tadpoles. Our results suggest that PFBA and PFHxA may have effects at environmentally-relevant concentrations (0.1–10 µg/L) and further investigation is required before these compounds can be deemed a “safe” alternative to their long-chain counterparts.

## Introduction

Per- and poly-fluoroalkyl substances (PFAS) are a class of > 4000 human-made compounds characterized by the presence of one or more perfluoroalkyl moieties (–C_n_F_2n+1_) (OECD 2013). PFAS function as surfactants (Buck et al. 2011) and the use of PFAS globally for both industrial and consumer applications is popular due to their chemical and thermal stability, and insulating and low surface energy properties (OECD 2013). Perfluoroalkyl carboxylic acids (PFCAs) are a group of PFAS that have received a lot of attention due to their persistence, bioaccumulative properties, and toxicity. The most commonly detected PFCA in the environment is perfluorooctanoic acid (PFOA) (Ankley et al. 2021). PFOA is a long-chain PFCA (a PFCA with ≥7 perfluoroalkyl carbons) with a high elimination half-life and a persistent and bioaccumulative nature (OECD 2013). Furthermore, PFOA is hazardous to humans and wildlife (Teaf et al. 2019). Manufacturers have phased out the use of PFOA since the early 2000s due to its known toxicity and its persistent nature (Lindstrom et al. 2011; OECD 2013). However, alternative short-chain (≤6 perfluoroalkyl carbons) PFCAs have been introduced into industrial use since the phasing out of PFOA. Subsequently, researchers have detected these alternative short-chain PFCAs in the environment (Houde et al. 2011; Li et al. 2020).

Short-chain PFCAs enter the environment via industrial emissions, disposal of PFCA-containing products, wastewater effluent discharge, and degradation of short-chain PFCA precursors (Brendel et al. 2018; Stoiber et al. 2020). All PFCAs, including short-chain counterparts, are known to be environmentally persistent due to their resistance to biotic and abiotic degradation (Brendel et al. 2018). In addition, short-chain PFCAs are highly mobile in soil and water leading to their ubiquitous spread globally (Brendel et al. 2018). Environmental surveys have reported the presence of short-chain PFCAs globally in river water, seawater, groundwater, drinking water, and the atmosphere (Li et al. 2020). The reported concentrations of short-chain PFCAs in aquatic systems ranged from 0.01 to 6280 ng/L (Li et al. 2020). The ubiquitous spread and the persistence of these compounds provide the potential for wildlife exposure, and recent studies suggest that short-chain PFCAs may exhibit toxic effects on wildlife. For example, chronic toxicity testing using zebrafish (*Danio reiro*) showed that exposure to short-chain PFCAs alters swimming behaviour in juvenile fish at concentrations of 10–100 mg/L (Menger et al. 2020). Further, embryonic exposure to 137 mg/L perfluorobutanoic acid (PFBA) altered growth rates and swim bladder physiology in Japanese medaka (*Oryzias latipes*) hatchlings (Godfrey et al. 2019). Additionally, researchers have detected short-chain PFCAs in the tissue of wildlife (Muir et al. 2019), indicating that these compounds may bioaccumulate and potentially result in prolonged exposure of organisms to toxic concentrations of the compounds.

The discharge of PFCAs into the aquatic environment may be a potential hazard to organisms such as amphibians, which have sensitive aquatic embryonic and larval stages that may be continuously exposed to aquatic contaminants via thin/permeable skin (Bentley and Yorio 1976). Amphibians are currently facing significant and widespread declines and are the most threatened vertebrate class globally (IUCN 2023); yet aquatic toxicity studies still focus primarily on fish. However, the study of amphibians can provide essential information regarding the toxicity of environmental contaminants. For example, the amphibian metamorphic assay can detect possible endocrine-disrupting properties of chemical groups (OECD 2009). In addition, amphibians have important ecological roles in aquatic ecosystems as both primary and secondary consumers (dependent on life-history stage). Previous research has linked amphibian population declines to significant changes in ecosystem function and structure, including changes to consumer populations, altered algal community structure, reduced nutrient cycling, and reduced energy transfer between aquatic and terrestrial habitats (Whiles et al. 2006; Whiles et al. 2013). Therefore, researchers can use amphibian toxicity studies to assess the potential influence of environmental contaminants on ecosystem health and function. We investigated the chronic toxicity and the bioconcentration of two short-chain PFCAs, perfluorobutanoic acid (PFBA) and perfluorohexanoic acid (PFHxA), using northern leopard frog (*Rana pipiens*) tadpoles. We selected these two compounds for testing because they are among the most commonly detected short-chain PFCAs in the aquatic environment (Li et al. 2020). Overall, our research will provide insight into whether replacement short-chain PFCAs are safer alternatives to their long-chain counterparts. Additionally, our research will provide data for future risk assessment of the tested compounds.

## Methods

### Animal collection and husbandry

Egg masses from wild northern leopard frogs (*Rana pipiens*) collected following standard artificial breeding protocols (Trudeau et al. 2013; Vu et al. 2017) were obtained from the University of Ottawa. Egg masses were transported to Carleton University (Ottawa, ON), and placed into 60-L aerated plastic stock tanks filled with dechloraminated (using Prime^®^ conditioner) city of Ottawa tap water aged for 48–72 h in a climate-controlled Conviron^®^ environmental chamber (temperature: 23 °C; humidity: 50%; diurnal cycle: 16 h light: 8 h dark). Stock colonies were monitored for their health status (and hatching) daily, with 50% water changes occurring thrice weekly. After the eggs had hatched, we provided larvae President’s Choice^®^ frozen kale and Wards *Xenopus* tadpole food *ad libitum* at the time of the water changes; we fed 1–3 algae wafers (Hikari KYORIN Co Inc.) once per week to supplement feeding. We measured water quality parameters once per week to ensure optimal growth conditions in accordance with published guidelines (OECD 2009).

### PFHxA and PFBA chronic amphibian toxicity test

Approximately three weeks after receiving the egg masses, we selected Gosner stage 25 (GS25) tadpoles (Gosner 1960) for chronic (43–46 d) toxicity testing. Tadpoles were exposed to either perfluorobutanoic acid (PFBA; CAS 375-22-4, Sigma Aldrich, Lot# STBH4094, Purity = 98%) or perfluorohexanoic acid (PFHxA; CAS 307-24-4, Toronto Research Chemicals, Canada, Lot#: 1-MX2-23-1, Purity = 95%) with static-renewal in aerated 9-L plastic type A aquariums. Nominal exposure concentrations for each compound were 0, 0.1, 1, 10, 100 and 1000 μg/L. PFBA has been detected in surface waters at concentrations up to 6.18 µg/L and PFHxA has been detected at lower concentrations (up to 0.462 µg/L) (Li et al. 2020). Therefore, our exposure concentrations incorporated environmentally relevant concentrations (0.1–10 µg/L). The higher exposure concentrations (100 µg/L and 1000 µg/L) were included to emulate worst-case scenarios for risk assessment purposes. We prepared the PFBA stock solutions by dissolving 36.5 µL of PFBA (density = 1.645 g/mL) in 1 L of distilled water, followed by serial dilutions for final stock solution concentrations of 0.006, 0.06, 0.6, 6, and 60 mg/L. We prepared the PFHxA stock solutions by dissolving 34.1 µL of PFHxA (density = 1.759 g/mL) in 1 L of distilled water, followed by serial dilutions for final stock solution concentrations of 0.006, 0.06, 0.6, 6, and 60 mg/L. Stock solutions were prepared every two weeks and stored at 4 ^o^C. The controls consisted of dechloraminated tap water aged > 48 h. Initial aquaria set-up consisted of 3 L of stock tadpole water (to reduce osmotic shock upon initial introduction to the treatment tank) and 3 L of aged dechloraminated water. The ten chemical treatments (five concentrations each for PFBA and PFHxA) had four replicate tanks per treatment, and the control had five replicate tanks (45 tanks total). The position of the tanks within the environmental chamber was assigned using a randomized complete block design; we placed one replicate tank per treatment in each of the four blocks (Block 4 had two control tanks). We selected 11 GS25 tadpoles in a random/unsystematic fashion from stock tanks, photographed them for biometrics, assessed them for health (normal body shape/no morphological abnormalities, and normal swimming behaviour), and randomly added them to each experimental tank within a replicate block, starting with replicate 1. We left the tadpoles to acclimatize for 2 h; we then dosed the tanks with 100 mL of the respective stock solutions to reach the desired nominal exposure concentrations. We collected water samples 1 h after dosing to establish initial exposure concentrations from the control, and the 0.1 µg/L, 10 µg/L, and 1000 µg/L PFBA and PFHxA treatments. Ninety-six hours after the initial set-up, we removed one tadpole from each tank, and we photographed, weighed, euthanized, flash froze in liquid nitrogen, and stored them at −80 °C for a separate study. Therefore, our initial sample size was 450 tadpoles for the chronic exposure study.

We performed 50% water changes (with compound renewal) two times a week (Mondays and Thursdays). As part of this process, we siphoned organic waste from the tanks to maintain water quality. We measured water quality parameters (temperature, pH, dissolved oxygen, and conductivity) once a week prior to the water changes, using a YSI Professional Plus meter (YSI Inc., Yellow Springs, OH, USA). We assessed ammonia, nitrate, nitrite, and water hardness using API® Freshwater Master Test Kits and API® GH and KH Test Kits (Mars Fishcare North America Inc.). Given the known stability of PFCAs, we did not expect breakdown to occur and therefore only re-inoculated replacement water. We monitored tanks daily for tadpole survival and health. We removed tadpoles that reached GS42 prior to the end of the experiment from the tanks and processed them as described below.

### Amphibian assessment

Following 43–46 days of exposure, we removed all surviving tadpoles from the tanks for final endpoint assessments; we processed one replicate tank per treatment per day (4 days total). We anesthetized each tadpole in a 0.02% solution of buffered MS-222. We weighed (Mettler Toledo Model: MS204TS) and photographed (Sony Cyber-Shot Digital Camera DSC-RX100) each tadpole for later biometric measurements. We then euthanized tadpoles in 0.20% MS-222, and determined their Gosner stage of development (Gosner 1960). We excised, weighed, and preserved the liver in liquid nitrogen, then stored the liver at −80 ^°^C for later bioconcentration analysis. We removed and discarded the stomach and intestines to prevent gut contents from negatively influencing body bioconcentration measurements and re-weighed the tadpole (Mettler Toledo Model: MS204TS). We determined tadpole sex morphologically using a dissection microscope (ZEISS Stemi 508) (Fig. S1). We froze (−40 °C) all tadpoles and later randomly sub-sampled from the control and the 1000 µg/L PFBA and 1000 µg/L PFHxA treatments for bioconcentration analysis (*n* = 3 per treatment).

We measured snout-to-vent length (SVL), body width, and tail length of each tadpole from the individual photographs using the imaging software ImageJ (version 1.52a; Wayne Rasband, National Institutes of Health, USA). To reduce observer bias, the same observer performed all biometric measurements. The observer took repeated measurements (*N* = 20) of three tadpoles throughout this procedure without reference to previous measurements; the mean coefficients of variation on repeated measurements ranged from 0.01 to 0.02%.

### Chemical analysis

Aqueous media and tadpole tissues were analyzed for PFBA and PFHxA at Environment and Climate Change Canada in Burlington, ON using previously published methods (Bartlett et al. 2021; de Solla et al. 2012). The sampling strategy was designed to reduce the analytical sample load whereby samples (pooled by treatment) were analyzed from 5 time points (Day 0, 3, 24, 28, and 42; with Day 0, 24 and 42 water samples collected 1 h after water changes/compound renewal and Day 3 and 28 before water changes/compound renewal) for the lowest and highest treatments, 0.1 and 1000 µg/L for each chemical. Control media was sampled on Day 0 and Day 42 and the intermediate treatment of 10 µg/L was analyzed on Day 0.

Care was taken to reduce any background PFAS contamination, which was monitored using laboratory blanks. For lab blanks, control media samples, and samples from treatments with nominal concentrations of PFCAs < 50 µg/L, samples were reconstituted in 1:1 methanol aqueous composition and spiked with 30 µL isotopically labeled standard to reach a final concentration of 1 ng/ml ^13^C_4_-PFBA and ^13^C_4_-PFHxA. For higher-concentration treatments, a dilution was required to bring the concentration to the linear range of the instrument. Thus, media from 1000 µg/L exposure treatments was subjected to a 100-fold dilution whereby 100 µL of media was diluted with 10 mL of methanol in a 15 mL centrifuge tube. A 0.25 mL subsample was combined with 30 μL isotopically labeled internal standard and 220 μL methanol prior to vialing for instrumental analysis.

Subsamples of carcass homogenate and liver (0.2–0.3 g) were extracted after spiking the isotopically labeled standard and combining with 5 mL of acetonitrile. The sample was vigorously agitated through a combination of vortex, ultrasonication and rotary shaking. The acetonitrile phase was isolated after centrifugation and the procedure was repeated with a second aliquot of acetonitrile. The combined extract was concentrated to 1 mL and then further polished using 25 mg of ENVI-carb™ (graphitized non-porous carbon). The supernatant was concentrated to 0.5 mL using nitrogen gas and combined with 0.5 mL of polished HPLC-grade water (polished using weak anion exchange solid phase extraction). In addition to method blanks, QA/QC included spike and recovery experiments and analysis of NIST SRM 1947 Lake Michigan fish tissue. Recovery of spiked analytes and agreement with the reference value was 88–99%.

All media and extracted biota samples were analyzed for PFCAs using ultra high-performance liquid chromatography (Acquity i-class, Waters Corporation) tandem mass spectrometry (Acquity TQS, Waters Corporation) (UHPLC-MS/MS) in electrospray negative ionization mode. A 15-level calibration curve (0.01–30 ng/mL) was used to quantify the concentration of analyte based on relative response to the internal standard, which inherently corrects for any matrix effects or recovery. Lab blanks were free of analytes. Detection limits were based on the instrumental detection limit - a concentration of analyte yielding a signal to noise ratio of 3, which corresponded to 0.006 ng/mL PFBA and 0.005 ng/mL PFHxA.

### SMI calculation

We calculated Scaled Mass Index (SMI) values for each tadpole using the following equation:

$${\widehat{M}}_{i}={M}_{i}{\left[\frac{{L}_{0}}{{L}_{i}}\right]}^{{b}_{SMA}}$$

Where *M*_*i*_ and *L*_*i*_, are the body mass and SVL of the individual tadpole *i*, respectively; *L*_0_ is the calculated mean SVL of all tadpoles in the dataset; and *b*_*SMA*_ is the scaling coefficient obtained from a standardised major axis (SMA) regression of ln mass versus ln SVL (Peig and Green 2009). We conducted the SMA regression using the ‘sma()’ function from the ‘smatr’ R package (Warton et al. 2012). We only used data from the control tadpoles to determine the scaling coefficient (*b*_*SMA*_) to ensure that treatment effects did not influence the coefficient; *b*_*SMA*_ = 2.542. We used data from all tadpoles included in the respective datasets to calculate mean SVLs (following Abercrombie et al. 2021; Flynn et al. 2022); PFBA dataset: *L*_0_ = 21.583, PFHxA dataset: *L*_0_ = 22.030.

### Statistical analysis

We conducted all analyses in R Studio 2022.12.0 using R 4.2.2 (R Core Team 2022). We used General Linear Mixed Models (GLMMs) with a Gaussian distribution, fitted using maximum likelihood, to assess the effects of PFCA exposure on tadpole snout-to-vent length (SVL), mass, scaled mass index (SMI), and hepatic-somatic index (HSI) using the ‘lme4’ package (Bates et al. 2015). For each compound, we compared response variables of the PFCA treatments to the controls. For all models, we checked the assumption of normality via visual inspection of histograms of the residuals, we plotted residuals against fitted values and against each explanatory variable to assess homogeneity of variances and independence (Zuur et al. 2009). Finally, we verified that the response variable was a reasonably linear function of the fitted values. We included tank as random effect to account for the non-independence of tadpoles in the same tank. We did not include block as a random effect since we found no difference in abiotic measures among treatments (Tables S1 & S2). Treatment and Gosner stage of development were included as fixed effects. We included SVL as an additional fixed effect in the models that assessed treatment effects on tadpole mass (Models 3 & 4). We selected the best-fit model by removing fixed effects using backwards selection and then comparing the Akaike Information Criterion (AIC) values (Nakagawa and Cuthill 2007) (Table S3). We log-transformed the response variables mass, SMI, and HSI to meet the assumption of normality for Models 3 to 8. In addition, when we plotted the response variables of SVL (Models 1 & 2), tadpole mass (Models 3 & 4) and SMI (Models 5 & 6) versus the fixed effect of Gosner stage we noticed the potential for a higher-order relationship in all cases. Therefore, we re-ran models 1 to 6 using a third order polynomial term for Gosner stage (Figs. S2–S7). Finally, the model examining PFHxA exposure effects on tadpole SVL (Model 2) would not converge due to low variability among tanks (Fig. S8). Therefore, we removed tank as a random effect and conducted a general linear model (GLM) with treatment and stage as predictor variables.

For each compound, we assessed treatment effects on Gosner stage using the Multiquantal Jonckheere-Terpstra (MQJT) test following the steps outlined in Green et al. (2018). We calculated the 20th, 30th, 40th, 50th, 60th, 70th, and 80th percentiles of Gosner stage of development for each replicate of each treatment. We then performed JT trend tests using the ‘clinfun’ package (Venkatraman and Whiting 2022) for each percentile, which compared the differences in percentile values among treatments, and recorded the p-values for each test. We then determined the median *p*-value for all p-values from the JT trend tests. The median *p*-values exceeded 0.05 for both compounds, indicating that the No Observed Effect Concentrations (NOECs) exceeded the highest tested values, and therefore we took no further steps in the analyses (Green et al. 2018).

For each compound, we conducted a General(ized) Linear Model (GLM) with a binomial distribution and logit link function to describe treatments effects on tadpole sex. We only used data from tadpoles that had developed to, or beyond, Gosner stage 36, once gonads are morphologically distinct (Hogan et al. 2008).

We calculated tadpole liver bioconcentration factors (BCFs) by dividing the wet weight tissue concentration (µg/kg w.w.) of the given compound (PFBA or PFHxA) by the average measured water concentration (µg/L) for a final BCF in units of L/kg. To calculate the whole-body burden of each compound we combined the measured wet weight liver and carcass concentrations (µg/kg w.w.) for each tadpole. We then calculated tadpole whole-body BCFs by dividing the whole-body burden (µg/kg w.w.) of the given compound (PFBA or PFHxA) by the average measured water concentration (µg/L) for a final BCF in units of L/kg (w.w.).

## Results

### Water quality parameters and measured PFBA and PFHxA concentrations

We maintained water quality parameters within suggested guidelines for amphibian testing (Table S4) (OECD 2009). In addition, we found no difference in mean water quality parameters among treatments (Tables S1 & S2). Measured exposure concentrations of PFBA and PFHxA ranged from 84.5 to 140% and 74 to 120% of nominal concentrations, respectively (Table S5). We detected cross-contamination of compounds in the PFBA 0.1 µg/L treatment early in the exposure but the PFHxA concentrations were below the limit of detection by Day 24. We detected minor contamination again on Day 28 with concentrations returning to below the limit of detection by Day 42. We detected low concentrations of PFBA in the control, PFHxA 0.1 µg/L, and PFHxA 10 µg/L treatments at various times throughout the experiment; however, the contamination was not persistent (Table S5).

### Amphibian growth and development

Tadpole survival was > 98% in all treatments. Tadpoles exposed to 0.1 to 100 µg/L of PFCAs were larger than control tadpoles. Specifically, tadpoles exposed to 0.1 and 10 µg/L of PFHxA had a significantly higher mean SVL than control tadpoles (Table 1). In addition, tadpoles exposed to 0.1, 1, 10, and 100 µg/L of PFBA, and tadpoles exposed to 0.1, 1, and 10 µg/L of PFHxA had a significantly higher mean mass (conditioned upon SVL and Gosner stage) than control tadpoles (Table 2). Tadpoles exposed to 0.1, 1, 10, and 100 µg/L of PFBA, and to 0.1, 1, and 10 µg/L of PFHxA had a significantly higher SMI than control tadpoles (Table 3). We found no effect of PFCA exposure on tadpole Gosner stage of development; the median *p*-values for Multiquantal Jonckheere-Terpstra (MQJT) test were 0.81 and 0.14 for PFBA and for PFHxA, respectively. Therefore, the NOEC exceeded the highest treatment concentrations. We found no effect of PFCA exposure on tadpole hepatic-somatic index (Table S6). Tadpoles exposed to 1 µg/L of PFBA were more likely to be male compared to control tadpoles (OR = 3.25, *p* = 0.028, Table S7).

**Table 1:** Results from models examining differences in snout-to-vent length (SVL) (mm) between exposure treatments and the control (intercept) in the chronic exposure study of *Rana pipiens* tadpoles exposed to PFBA (GLMM) and PFHxA (GLM)

PFBA									
		*n*	ß coef	SE	df	*t*-value	*p*-value	Variance	SD
**Model 1: SVL ~ Treatment + Stage + Stage ^ 2 + Stage ^ 3** + **(1 | Tank),** ***n*** = **247**									
Fixed Effects	(Intercept - Control)	50	329.310	57.568	242.2	5.720	**<** **0.001**		
	0.1 µg/L	40	0.024	0.364	23.6	0.067	0.947		
	1 µg/L	38	0.395	0.368	24.7	1.074	0.293		
	10 µg/L	39	−0.069	0.363	23.6	−0.190	0.851		
	100 µg/L	40	0.198	0.362	23.1	0.548	0.589		
	1000 µg/L	40	0.454	0.361	23.0	1.258	0.221		
	Stage		−28.709	4.849	242.1	−5.921	**<** **0.001**		
	Stage^2		0.844	0.135	241.9	6.244	**<** **0.001**		
	Stage^3		−0.008	0.001	241.8	−6.353	**<** **0.001**		
Random Effects	Tank							0.061	0.247
	Residual							2.287	1.512

PFHxA						
		*n*	ß coef	SE	*t*-value	*p*-value
**Model 2: SVL ~ Treatment + Stage + Stage ^ 2 + Stage ^ 3,** ***n*** = **246**						
Predictors	(Intercept - Control)	50	144.312	44.921	3.213	**0.001**
	0.1 µg/L	40	0.768	0.353	2.173	**0.031**
	1 µg/L	38	0.266	0.354	0.753	0.452
	10 µg/L	39	1.362	0.356	3.822	**<** **0.001**
	100 µg/L	40	0.266	0.351	0.759	0.449
	1000 µg/L	40	0.327	0.356	1.760	0.080
	Stage		−13.178	3.858	−3.416	**<** **0.001**
	Stage^2		0.414	0.109	3.786	**<** **0.001**
	Stage^3		−0.004	0.001	−3.905	**<** **0.001**

Model 1, treatment and Gosner stage of development (Stage) were included as fixed effects, and tank was included as a random effect. Model 2, treatment and Gosner stage of development (Stage) were included as predictors. Significant results are in bold

**Table 2:** Results from GLMMs examining differences in log-transformed mass (g) between exposure treatments and the control (intercept) in the chronic exposure study of *Rana pipiens* tadpoles exposed to PFBA and PFHxA

		*n*	ß coef	SE	df	*t*-value	*p*-value	Variance	SD
**PFBA**									
**Model 3: logMass ~ Treatment** + **SVL + Stage + Stage ^ 2 + Stage ^ 3** + **(1 | Tank),** ***n*** = **247**									
Fixed Effects	(Intercept - Control)	50	9.273	1.869	230.2	4.963	**<** **0.001**		
	0.1 µg/L	40	0.042	0.018	24.8	2.374	**0.026**		
	1 µg/L	38	0.049	0.018	25.4	2.747	**0.011**		
	10 µg/L	39	0.043	0.018	24.8	2.424	**0.023**		
	100 µg/L	40	0.037	0.018	24.6	2.068	**0.049**		
	1000 µg/L	40	0.021	0.018	24.7	1.151	0.261		
	SVL		0.051	0.002	237.7	25.636	**<0.001**		
	Stage		−0.996	0.158	230.2	−6.303	**<0.001**		
	Stage^2		0.031	0.004	230.3	7.077	**<0.001**		
	Stage^3		−3.21 × 10^−4^	4.108 × 10^−5^	230.4	−7.815	**<0.001**		
Random Effects	Tank							< 0.001	0.022
	Residual							0.002	0.045
**PFHxA**									
**Model 4: logMass ~ Treatment** + **SVL + Stage + Stage ^ 2 + Stage ^ 3** + **(1 | Tank),** ***n*** = **246**									
Fixed Effects	(Intercept - Control)	50	5.830	1.450	231.4	4.021	**<** **0.001**		
	0.1 µg/L	40	0.062	0.022	25.1	2.833	**0.009**		
	1 µg/L	39	0.056	0.022	24.9	2.568	**0.017**		
	10 µg/L	38	0.052	0.022	25.8	2.366	**0.026**		
	100 µg/L	40	0.024	0.022	24.7	1.102	0.281		
	1000 µg/L	39	0.039	0.022	25.2	1.774	0.088		
	SVL		0.050	0.002	230.0	24.354	**<** **0.001**		
	Stage		−0.702	0.125	231.1	−5.628	**<** **0.001**		
	Stage^2		0.023	0.004	230.9	6.493	**<** **0.001**		
	Stage^3		−2.426 × 10^−4^	3.336 × 10^−5^	230.8	−7.273	**<** **0.001**		
Random Effects	Tank							< 0.001	0.028
	Residual							0.003	0.050

Treatment, snout-to-vent length (SVL) (mm), and Gosner stage of development (Stage) were included as fixed effects, and tank was included as a random effect. Significant results are in bold

**Table 3:** Results from GLMMs examining differences in log-transformed scaled mass index (SMI) between exposure treatments and the control (intercept) in the chronic exposure study of *Rana pipiens* tadpoles to PFBA and PFHxA

		*n*	ß coef	SE	df	*t*-value	*p*-value	Variance	SD
**PFBA**									
**Model 5: logSMI ~ Treatment + Stage + Stage ^ 2 + Stage ^ 3** + **(1 | Tank),** ***n*** = **247**									
Fixed Effects	(Intercept - Control)	50	12.120	1.770	232.6	6.848	**<** **0.001**		
	0.1 µg/L	40	0.049	0.017	24.9	2.918	**0.007**		
	1 µg/L	38	0.050	0.017	25.4	2.977	**0.006**		
	10 µg/L	39	0.041	0.017	24.8	2.444	**0.022**		
	100 µg/L	40	0.037	0.017	24.6	2.224	**0.036**		
	1000 µg/L	40	0.022	0.017	24.6	1.282	0.212		
	Stage		−1.100	0.149	232.5	−7.379	**<** **0.001**		
	Stage^2		0.033	0.004	232.4	8.001	**<** **0.001**		
	Stage^3		−3.296 × 10^−4^	3.836 × 10^−5^	232.3	−8.592	**<** **0.001**		
Random Effects	Tank							< 0.001	0.020
	Residual							0.002	0.046
**PFHxA**									
**Model 6: logSMI ~ Treatment + Stage + Stage ^ 2 + Stage ^ 3** + **(1 | Tank),** ***n*** = **246**									
Fixed Effects	(Intercept - Control)	50	9.063	1.372	231.6	6.605	**<** **0.001**		
	0.1 µg/L	40	0.064	0.021	24.8	3.031	**0.006**		
	1 µg/L	39	0.054	0.021	24.9	2.539	**0.018**		
	10 µg/L	38	0.053	0.021	25.1	2.493	**0.020**		
	100 µg/L	40	0.023	0.021	24.7	1.066	0.297		
	1000 µg/L	39	0.036	0.021	25.0	1.688	0.104		
	Stage		−0.837	0.118	231.4	−7.108	**<** **0.001**		
	Stage^2		0.026	0.003	231.2	7.721	**<** **0.001**		
	Stage^3		−2.589 × 10^−4^	3.126 × 10^−5^	231.1	−8.281	**<** **0.001**		
Random Effects	Tank							0.001	0.028
	Residual							0.002	0.049

Treatment and Gosner stage of development were included as fixed effects, and Tank was included as a random effect. Significant results are in bold

### Bioconcentration

**Fig. 1:**
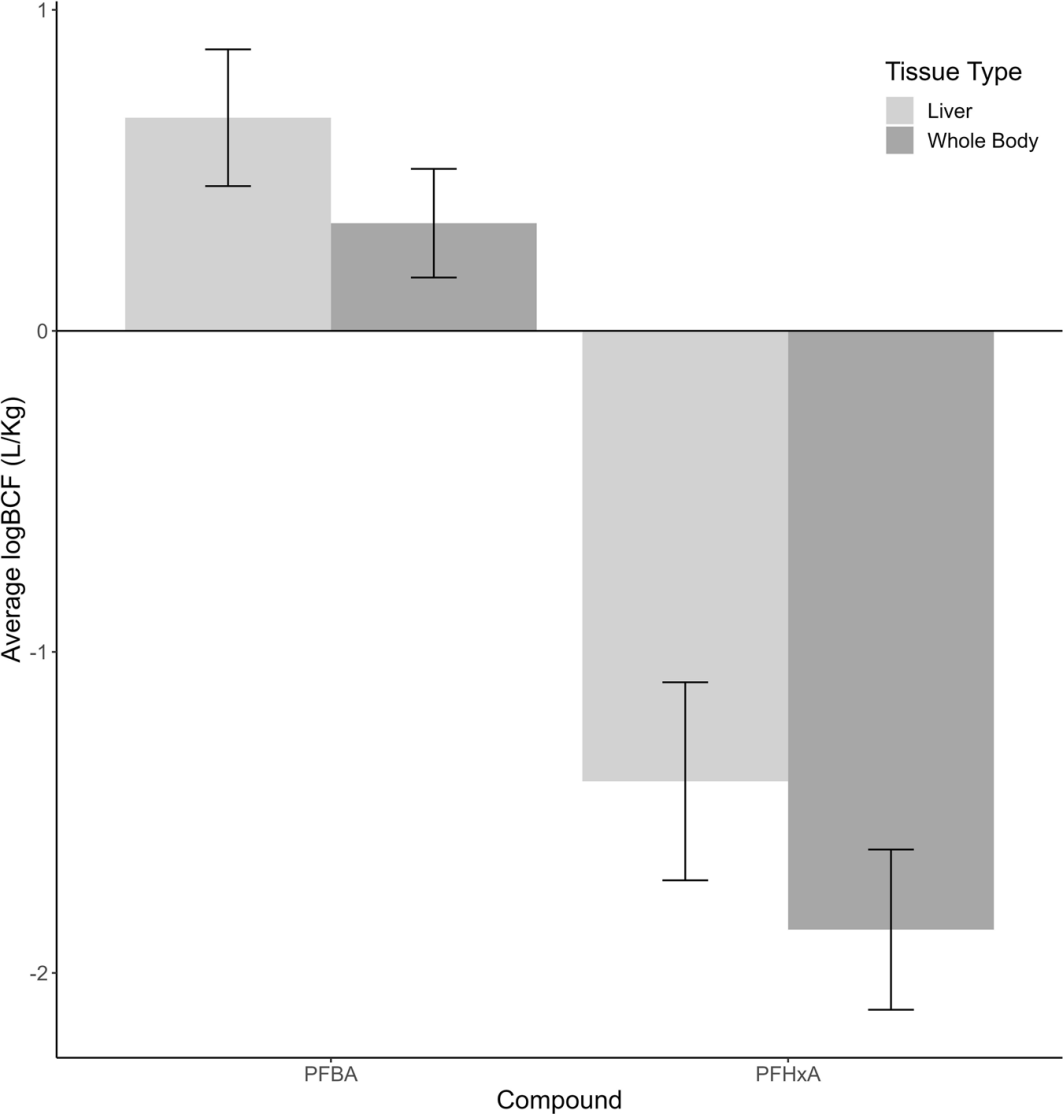
Average log bioconcentration factor (BCF) after 43–46 days of exposure to a nominal concentration of 1000 µg/L perfluorobutanoic acid (PFBA) or 1000 µg/L perfluorohexanoic acid (PFHxA) calculated for tadpole liver and whole-body samples. Error bars correspond to standard deviation

We calculated bioconcentration factors separately for whole-body and liver samples from the 1000 µg/L PFBA and the 1000 µg/L PFHxA treatments. Concentrations of PFBA detected in whole-body tadpole samples ranged from 1374 to 2966 µg/kg w.w., whereas concentrations of PFHxA were much lower, ranging from 7.78 to 23.53 µg/kg w.w. (Fig. 1, Table S8). The average ± SD BCF for PFBA in whole-body tadpole samples was 2.28 ± 0.91 L/kg w.w., 100-fold higher than the BCF for PFHxA (0.02 ± 0.01 L/kg). Concentrations of PFBA detected in tadpole liver samples ranged from 2493 to 6629 µg/kg w.w., up to five-fold higher than in whole-body samples. Concentrations of PFHxA detected in tadpole liver samples ranged from 21 to 80 µg/kg w.w., up to 10-fold higher than in whole-body samples but approximately 100-fold lower than PFBA. The average ± SD BCF for PFBA in liver samples was 4.98 ± 2.30 L/kg w.w., 100-fold higher than that for PFHxA (0.05 ± 0.04 L/kg w.w.). The liver to whole-body concentration ratio ± SD was similar between PFCAs at 2.6 ± 2.0 L/kg w.w. for PFBA and 3.0 ± 0.87 L/kg w.w. for PFHxA.

## Discussion

We observed that tadpoles exposed to 0.1–100 µg/L of the two short-chain PFCAs were significantly larger than control tadpoles. Although no studies on the effects of PFBA and/or PFHxA exposure on amphibian growth have been published to our knowledge, researchers have reported similar findings for other species. For example, dietary exposure to low (10–100 ng/L) concentrations of PFBA increased weight gain of beet armyworm (*Spodoptera exigua*) larvae (Omagamre et al. 2020). Similar effects have also been noted at higher exposure concentrations, for example exposure to 1 mg/L of PFBA and 1 mg/L of PFHxA (separately) increased body size and egg size of the rotifer *Brachionus calyciflorus* (Wang et al. 2014). In addition, female hatchlings of Japanese medaka (*Oryzias latipes*) embryos exposed to 137 mg/L of PFBA were significantly larger than controls (Godfrey et al. 2019). Interestingly, sub-chronic exposure (30 d) of northern leopard frog tadpoles to PFOA (a long-chain counterpart of PFBA and of PFHxA) via spiked sediment (10, 100, 1000 µg/kg, dry wet) in mesocosms did not affect tadpole SVL or mass (Flynn et al. 2021). Dermal exposure to 80, 800, or 8000 µg/kg dry weight of PFOA in moss did not influence northern leopard frog tadpole SVL or mass after 30 d of exposure (Abercrombie et al. 2021). A more recent study suggested that exposure to 1000 µg/L of PFOA for 30 d reduced mass of northern leopard frogs by approximately 30% (Flynn et al. 2022). However, the same study showed that exposure to 1000 µg/L 2:6 FTS, a compound that produces PFBA and PFHxA as degradation products (Méndez et al. 2022; Wang et al. 2011), significantly increased tadpole mass (Flynn et al. 2022). Flynn et al. (2022) also noted significant variation in growth outcomes (Mass, SVL, SMI) among amphibian species and within species, with SMI being the most sensitive measure to PFAS exposure.

In addition to changes in SVL and mass, we observed a significant increase in SMI, a measure of body condition, in the 0.1 to 100 µg/LPFCA treatment tadpoles. Increased body condition is associated with increased fitness in amphibians (Reading 2007). Furthermore, an increase in SMI is associated with increased energy stores in amphibians (MacCracken and Stebbings 2012). However, the composition of energy stores can vary (MacCracken and Stebbings 2012); therefore, without body composition analyses we are unable to discern if the tadpoles are accumulating higher percentages of lipids, proteins, or lean mass which may have different fitness effects in amphibians. For example, lipids are essential for amphibian metamorphosis, acting as one of the primary energy sources (Wright et al. 2011; Zhu et al. 2020), with higher lipid stores linked to increased survival of amphibians post-metamorphosis (Scott et al. 2007). Therefore, further investigation into the body composition of tadpoles exposed to short-chain PFCAs is required to understand the fitness effects that the observed changes in size and SMI may have. Contrary to our results, sub-chronic exposure (30 d) to 10, 100, and 1000 µg/L of PFOA reduced northern leopard frog SMI by approximately 5–10% (Flynn et al. 2022). Interestingly, while exposure to 1000 µg/L of 2:6 FTS significantly increased tadpole mass, tadpoles exposed to 10, 100, and 1000 µg/L of 2:6 FTS had significantly reduced SMIs relative to unexposed tadpoles (Flynn et al. 2022). Therefore, our study suggests that short-chain PFCAs affect body condition in amphibians in ways not previously observed for their most-commonly-detected long-chain counterpart, PFOA. More research is needed to determine the mode of action and impact of these effects.

While the mechanism causing an increase in size and in body condition is unknown, potential explanations include that PFCAs mimic fatty acids (Luebker et al. 2002; Peng et al. 2013; Khazaee et al. 2021) and may disrupt lipid metabolism (Mahapatra et al. 2017; Mentor et al. 2020; Zhang et al. 2019). In addition, PFCAs may interfere with endocrine processes associated with metabolism and/or development such as changes to glucocorticoid levels (Mortensen et al. 2011), thyroid hormone levels (Zhang et al. 2022), and/or changes to expression of corticotrophin-releasing factor (Wang et al. 2022) in vertebrates. Further investigation is required to understand the underlying mechanism(s) of size and body condition differences between treatment and control tadpoles.

We observed a non-monotonic concentration response for effects on size and SMI of tadpoles for both short-chain PFCAs. Numerous toxicological studies have reported non-monotonic concentration responses (summarized in Vandenberg et al. 2012; Lagarde et al. 2015). There are several proposed mechanisms by which compounds exert non-monotonic effects including acting as endocrine-disrupters by mimicking natural hormones at low concentrations, or by directly or indirectly influencing hormone metabolism, uptake, production, and/or transport (Vanderberg et al. 2012; Lagarde et al. 2015). Alternatively, non-monotonic concentration responses may potentially be due to an overcompensation in response to a contaminant stressor at lower concentrations with inhibition of the response at higher exposure concentrations (Calabrese 2001). Further investigation into how PFBA and PFHxA exert non-monotonic effects in amphibians is required. However, the observed effects at exposure concentrations ranging from 0.1 µg/L to 100 µg/L in our study are noteworthy considering that some toxicological studies have concluded that these short-chain substances have low toxicity compared to their long-chain counterparts based on reported LC50s and EC50s ≥ 1 mg/L (e.g., Barmentlo et al. 2015; Ulhaq et al. 2013).

We observed a significantly higher proportion of male tadpoles in the 1 µg/L PFBA treatment than in the control. However, the M:F ratio (1:0.8) observed in the 1 µg/L PFBA treatment is similar to previously reported sex ratios for unexposed northern leopard frog tadpoles (Hogan et al. 2008). We determined the sex of the tadpoles via visual observation as a preliminary screening for potential sex-ratio distortion, although histological analyses would be required to confirm sex ratios and determine whether intersex tadpoles were present (Hogan et al. 2008). Thus, a more in-depth investigation into the effects of PFBA and of PFHxA on tadpole sex ratios needs to be conducted before any concrete conclusions can be made.

The BCFs calculated for PFBA and PFHxA were similar (< 10 L/kg w.w.) to those reported for PFOA bioconcentration in northern leopard frogs (Abercrombie et al. 2019; Hoover et al. 2017). The BCF must be greater than 1000 for a compound to be considered bioaccumulative (US EPA 2020), and neither PFBA nor PFHxA met that criterion. Interestingly, previous studies using fish have shown that PFCAs with longer carbon chains tend to have higher BCFs than PFCAs with shorter carbon chains (Menger et al. 2020; Wen et al. 2019), but we observed the opposite trend in the current study, with the BCF for PFBA being 100-fold higher than the BCF for PFHxA. Similarly, a study with crucian carp (*Carassius carassius*) showed that PFHxA had lower bioaccumulation factors (BAFs) than some shorter chain PFCAs and that PFBA had higher BAFs than several other longer-chain PFCAs (Shi et al. 2018). The measured BAFs for short-chain PFCAs were not associated with chain-length; thus, the bioaccumulation patterns of short-chain PFCAs could not be explained by the phospholipid partitioning mechanism previously used to explain the bioaccumulation patterns of long-chain PFCAs (Shi et al. 2018). Instead, current research supports protein-binding mediated mechanisms of bioaccumulation for PFCAs (Bischel et al. 2011; Ren et al. 2022; Shi et al. 2018). As a result, the affinity of PFBA and PFHxA for amphibian proteins is a priority for future research. Reported BCFs for PFHxA in zebrafish (*Danio rerio*) were two orders of magnitude higher than what we calculated for northern leopard frogs (Menger et al. 2020; Wen et al. 2019), suggesting potential differences between taxa in PFCA bioconcentration. Differences in BCFs for PFOA between amphibians and fish have been previously reported (Abercrombie et al. 2019; Hoover et al. 2017). These variations in bioconcentration between taxa may be due to differences in ability to eliminate PFCAs, or due to differences in composition of tissues sampled. In addition, differences may be due to the life-stage sampled; amphibian growth is non-linear and amphibians undergo significant tissue re-modelling during metamorphosis (Brown and Cai 2007), which may lead to changes in bioconcentration throughout development. For example, Hoover et al. (2017) measured significant decreases in body burdens of PFOA in northern leopard frog tadpoles over time and attributed the decrease to anatomical changes in tadpoles that occurred during development. Further research is required to understand the effects of amphibian metamorphosis/development on bioconcentration of PFCAs and to determine the mechanism(s) responsible for reported differences between taxa. Our findings indicated that PFBA and PFHxA concentrations in the liver were nearly three times higher than the whole-body concentrations. Preferential accumulation of PFCA in the liver over other organs has been previously reported in other organisms, including polar bear and fish (e.g., Greaves et al. 2012; Petre et al. 2023; Wen et al. 2019). PFCA liver accumulation may provide a rationale for reported hepatotoxicity of PFAS in frogs (Lin et al. 2022a; Lin et al. 2022b; Zhang et al. 2019).

We detected cross contamination of compounds in the PFBA 0.1 µg/L treatment early in the exposure period. We also do not have measured concentrations of compounds for the 1 µg/L and 100 µg/L PFBA and PFHxA treatments. Therefore, we cannot be certain that observed effects in these treatments were not affected by potential cross-contamination. However, PFHxA in the PFBA 0.1 ug/L treatment was below detection limits by Day 24. Reduction of contamination at later sampling points is likely due to removal of the compound during the frequent (twice a week) 50% water changes, with only the desired test compound being renewed at each water change through re-inoculation of replacement water. Furthermore, the reduction of cross contamination as the exposure progressed (along with the continued stability of the compounds of interest) indicates that the primary exposure was to the compounds of interest, and that short-term exposure to the cross contaminant is unlikely to cause any of the observed effects.

## Conclusion

In conclusion, exposure to PFBA and PFHxA at concentrations ranging from 0.1 µg/L to 100 µg/L increased tadpole growth (mass, SVL, and SMI) however, the mechanism behind this effect is unknown. Further investigation is required to understand the underlying mechanism(s) of size differences between treatment and control tadpoles. We observed a higher proportion of males in the PFBA 1 µg/L treatment group, however further histological analyses are required before making concrete conclusions on the effects of PFCAs on amphibian sex ratios. Our results suggest that PFBA and PFHxA are taken up by tadpoles and accumulate to higher concentrations in the liver than in whole-body samples, but overall bioconcentration factors are low and do not meet the threshold to be considered bioaccumulative. However, differences in trends observed in BCFs for PFBA and PFHxA in our study compared to fish studies (i.e., PFBA accumulating at higher concentrations than PFHxA in tadpoles, and overall lower BCFs for tadpoles than for fish) suggest potential differences between taxa in PFCA bioconcentration in aquatic ecosystems. Therefore, BCFs determined for one species may not necessarily reflect the overall trend of bioconcentration for all aquatic species. Overall, our study suggests that exposure to replacement short-chain PFCAs at environmentally-relevant concentrations may influence amphibian growth, and further investigation is required before these compounds can be deemed a “safe” alternative to their long-chain counterparts.

### Supplementary information

Supplementary Information
