# Supplementary material for: The effects of two short-chain perfluoroalkyl carboxylic acids (PFCAs) on northern leopard frog (Rana pipiens) tadpole development
Source: Ecotoxicology. 2024 Feb 5;33(2):177–89. doi: 10.1007/s10646-024-02737-z (PMC10940426; doi:10.1007/s10646-024-02737-z)
Supplement: Supplementary file 1 — Supplementary Information [file 10646_2024_2737_MOESM1_ESM.pdf]

Appendix A. Supplemental Information

The effects of two short-chain perfluoroalkyl carboxylic acids (PFCAs) on northern leopard frog (*Rana pipiens*) tadpole development

Jillian Rohonczy[a], Stacey A. Robinson[a,b*], Mark R. Forbes[a], Amila O. De Silva[c], Cassandra Brinovcar[c], Adrienne J. Bartlett[c], Ève A.M. Gilroy[c]

[a] Department of Biology, Carleton University, Ottawa, ON, K1S 5B6, Canada

[b] Wildlife and Landscape Science Directorate, Environment and Climate Change, Ottawa, ON K1A 0H3, Canada

[c] Water Science and Technology Directorate, Environment and Climate Change Canada, Burlington, ON, L7S 1A1, Canada

***Corresponding Author:** Stacey A. Robinson, email: Stacey.Robinson@ec.gc.ca, Tel: +1-343-998-3719

Contents

Fig. S1 Photographs of A) female and B) male gonads from *Rana pipiens* tadpoles sampled from the control group of the chronic exposure study with PFCAs. Both tadpoles are Gosner stage 40. Bar represents 0.05 mm. O; Ovary T; Testis, K; Kidney. 3

Table S1. Results from ANOVAs comparing mean water quality measures among treatment groups in the chronic PFCAs exposure study with *Rana pipiens* tadpoles. 4

Table S2. Results from the Kruskal-Wallis analyses median water quality measures among treatment groups in the chronic PFCAs exposure study with *Rana pipiens* tadpoles. 4

Table S3. General linear mixed model comparisons and Akaike Information Criterion (AIC) values for each predictor variable (fixed effects) combination. Predictor variables included exposure/control treatments (Treatment), snout-to-vent length (SVL), and Gosner stage (Stage) of northern leopard frog (*Rana pipiens*) tadpoles exposed to PFCAs in a chronic study. We included tank as a random effect in models to account for the non-independence of tadpoles in each tank. The best-fit AIC values are denoted by an asterisk (*). 5

Fig. S2 Scatterplot of *Rana pipiens* tadpole snout-to-vent length (SVL) (mm) by Gosner stage of development following chronic exposure to PFBA. Red line shows first order polynomial relationship. Blue line indicates second order polynomial relationship (Stage^2). Green line indicates a third order polynomial relationship (Stage^3). 6

Fig. S3 Scatterplot of *Rana pipiens* tadpole snout-to-vent length (SVL) (mm) by Gosner stage of development following chronic exposure to PFHxA. Red line shows first order polynomial relationship. Blue line indicates second order polynomial relationship (Stage^2). Green line indicates a third order polynomial relationship (Stage^3). 7

Fig. S4 Scatterplot of *Rana pipiens* tadpole mass (g) by Gosner stage of development following chronic exposure to PFBA. Red line shows first order polynomial relationship. Blue line indicates second order polynomial relationship (Stage^2). Green line indicates a third order polynomial relationship (Stage^3). . 8

Fig. S5 Scatterplot of *Rana pipiens* tadpole mass (g) by Gosner stage of development following chronic exposure to PFHxA. Red line shows first order polynomial relationship. Blue line indicates second order polynomial relationship (Stage^2). Green line indicates a third order polynomial relationship (Stage^3). . 9

Fig. S6 Scatterplot of *Rana pipiens* tadpole scaled mass index (SMI) by Gosner stage of development following chronic exposure to PFBA. Red line shows first order polynomial relationship. Blue line indicates second order polynomial relationship (Stage^2). Green line indicates a third order polynomial relationship (Stage^3). 10

Fig. S7 Scatterplot of *Rana pipiens* tadpole scaled mass index (SMI) by Gosner stage of development following chronic exposure to PFHxA. Red line shows first order polynomial relationship. Blue line indicates second order polynomial relationship (Stage^2). Green line indicates a third order polynomial relationship (Stage^3). 11

Fig. S8 Scatterplot of mean *Rana pipiens* tadpole snout-to-vent length (SVL) (mm) by treatment tank following chronic exposure to PFHxA highlighting the low variation in tadpole SVL among tanks. Treatment and replicate tank number are shown on the x-axis (i.e., Controls: T11-1 to T11-5, PFHxA 0.1 to 1000 ug/L: T6-1 to T10-4). Error bars correspond to standard deviation. 12

Table S4. Average water quality parameters (temperature (°C), dissolved oxygen (% and mg/L), pH, conductivity (µS/cm), ammonia (mg/L), nitrate (mg/L), nitrite (mg/L)), and water hardness (mg/L)), by

treatment taken throughout the chronic PFCA exposure study with Rana pipiens tadpoles. Data are reported as mean ± standard deviation. 13

Table S5. Measured concentrations of perfluorobutanoic acid (PFBA) and perfluorohexanoic acid (PFHxA) and percent nominal concentrations detected in treatment solutions throughout the chronic experiment with *Rana pipiens* tadpoles. Analysis conducted on pooled samples by treatment across replicates. Method detection limits are presented when no analytical peak was detected. The method detection limit is the instrumental detection limit multiplied by the dilution factor. Hyphen (-) indicates value not quantified. 14

Table S6. Results from GLMMs examining differences in log-transformed hepatic-somatic index (HSI) between exposure treatments and the control (intercept) in the chronic exposure study of *Rana pipiens* tadpoles to PFBA and PFHxA. Treatment and Gosner stage of development were included as fixed effects, and tank was included as a random effect. Significant results are in bold. 15

Table S7. Results from logistic regressions examining the probability of tadpoles being male between exposure treatments and the control (intercept) in the chronic exposure study of *Rana pipiens* tadpoles exposed to PFBA and PFHxA. Significant results are in bold. 16

Table S8. Bioconcentration factors (BCF) calculated using average measured exposure concentrations and measured wet weight (w.w.) whole-body and liver concentrations from the 1000 µg/L PFBA and the 1000 µg/L PFHxA treatments in the chronic exposure study of *Rana pipiens* tadpoles. Data are shown for 3 individual tadpoles and resulting average ± SD. 17

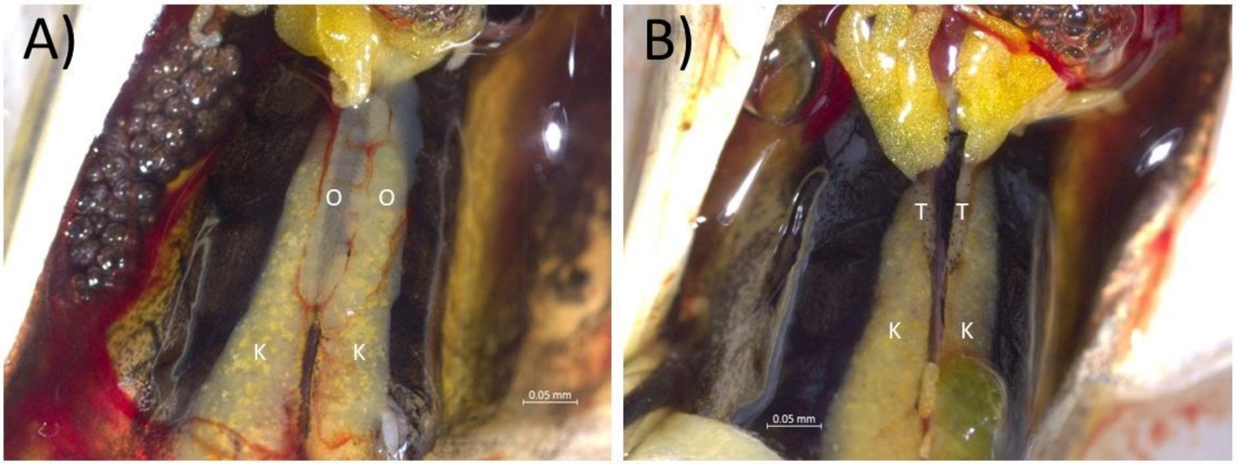

Fig. S1 Photographs of A) female and B) male gonads from *Rana pipiens* tadpoles sampled from the control group of the chronic exposure study with PFCAs. Both tadpoles are Gosner stage 40. Bar represents 0.05 mm. O; Ovary T; Testis, K; Kidney.

Table S1. Results from ANOVAs comparing mean water quality measures among treatment groups in the chronic PFCAs exposure study with *Rana pipiens* tadpoles.

Response Variable	Df	Sum Sq	Mean Sq	F-value	P-value
Temperature	10	0.765	0.077	1.397	0.201
Dissolved Oxygen	10	547	54.690	0.810	0.620
pH	10	0.721	0.072	1.308	0.245
Conductivity	10	7273	727.3	1.196	0.310
Ammonia	10	48.490	4.849	1.209	0.302

Table S2. Results from the Kruskal-Wallis analyses median water quality measures among treatment groups in the chronic PFCAs exposure study with *Rana pipiens* tadpoles.

Response Variable	Df	Chi-squared	P-value
Nitrate	10	6.656	0.757
Nitrite	10	7.715	0.657
Hardness	10	3.669	0.961

Table S3. General linear mixed model comparisons and Akaike Information Criterion (AIC) values for each predictor variable (fixed effects) combination. Predictor variables included exposure/control treatments (Treatment), snout-to-vent length (SVL), and Gosner stage (Stage) of northern leopard frog (*Rana pipiens*) tadpoles exposed to PFCAs in a chronic study. We included tank as a random effect in models to account for the non-independence of tadpoles in each tank. The best-fit AIC values are denoted by an asterisk (*).

Model	AIC
**Model 1: Snout-to-vent length (SVL) - PFBA**	
A. SVL ~ Treatment + Stage + (1|Tank)	971.035*
B. SVL ~ Treatment + (1|Tank)	1359.238
**Model 2: Snout-to-vent length (SVL) - PFHxA**	
A. SVL ~ Treatment + Stage	972.064*
B. SVL ~ Treatment	1312.626
**Model 3: Mass - PFBA**	
A. Mass ~ Treatment + SVL + Stage + (1|Tank)	164.864*
B. Mass ~ Treatment + SVL + (1|Tank)	197.770
C. Mass ~ Treatment + Stage + (1|Tank)	420.528
D. Mass ~ Treatment + (1|Tank)	605.203
**Model 4: Mass - PFHxA**	
A. Mass ~ Treatment + SVL + Stage + (1|Tank)	177.401*
B. Mass ~ Treatment + SVL + (1|Tank)	197.246
C. Mass ~ Treatment + Stage + (1|Tank)	415.793
D. Mass ~ Treatment + (1|Tank)	583.553
**Model 5: Scaled mass index (SMI) - PFBA**	
A. SMI ~ Treatment + Stage + (1|Tank)	59.032*
B. SMI ~ Treatment + (1|Tank)	62.842
**Model 6: Scaled mass index (SMI) - PFHxA**	
A. SMI ~ Treatment + Stage + (1|Tank)	109.720*
B. SMI ~ Treatment + (1|Tank)	109.970
**Model 7: Hepatic-somatic index (HSI) - PFBA**	
A. HSI ~ Treatment + Stage + (1|Tank)	660.768*
B. HSI ~ Treatment + (1|Tank)	842.196
**Model 8: Hepatic-somatic index (HSI) - PFHxA**	
A. HSI ~ Treatment + Stage + (1|Tank)	671.746*
B. HSI ~ Treatment + (1|Tank)	836.211

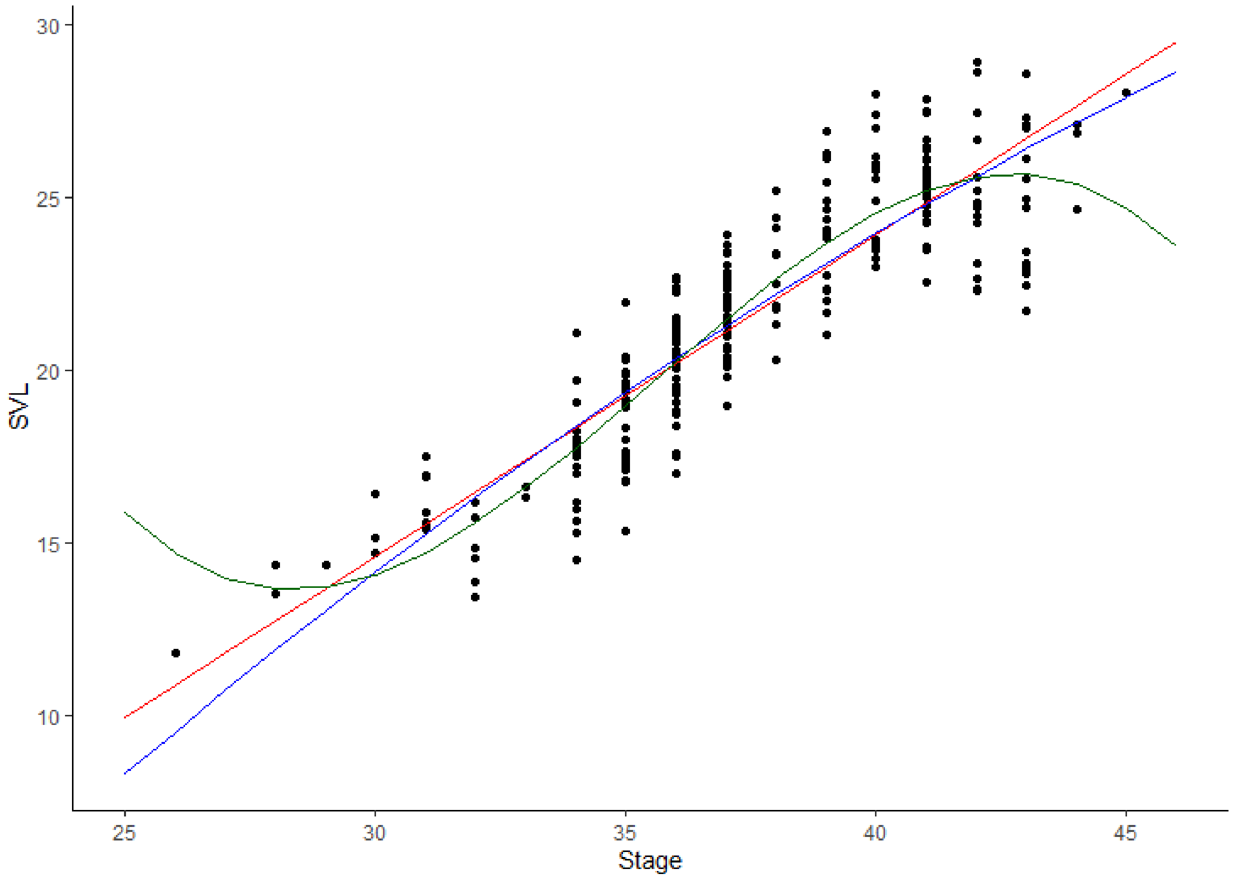

Fig. S2 Scatterplot of *Rana pipiens* tadpole snout-to-vent length (SVL) (mm) by Gosner stage of development following chronic exposure to PFBA. Red line shows first order polynomial relationship. Blue line indicates second order polynomial relationship (Stage^2). Green line indicates a third order polynomial relationship (Stage^3).

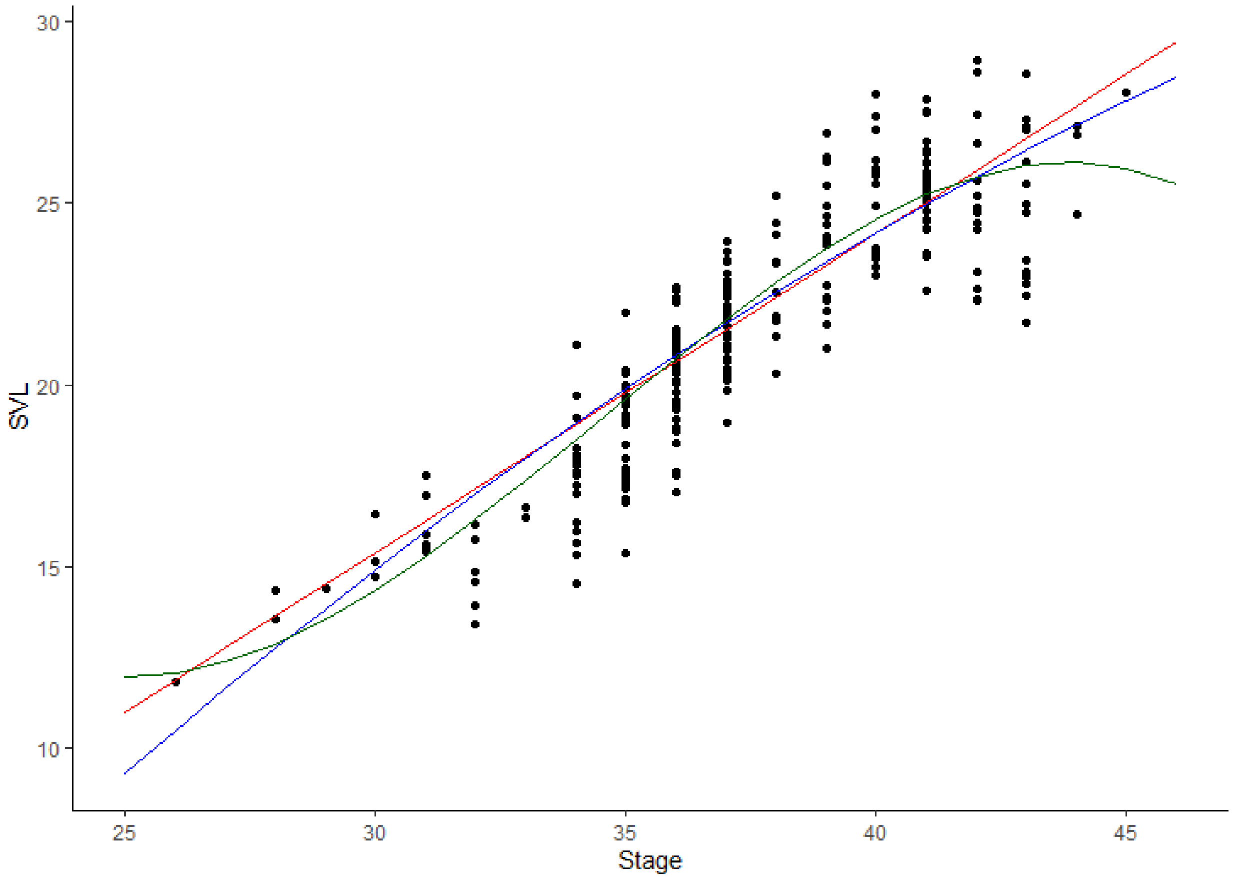

Fig. S3 Scatterplot of *Rana pipiens* tadpole snout-to-vent length (SVL) (mm) by Gosner stage of development following chronic exposure to PFHxA. Red line shows first order polynomial relationship. Blue line indicates second order polynomial relationship (Stage^2). Green line indicates a third order polynomial relationship (Stage^3).

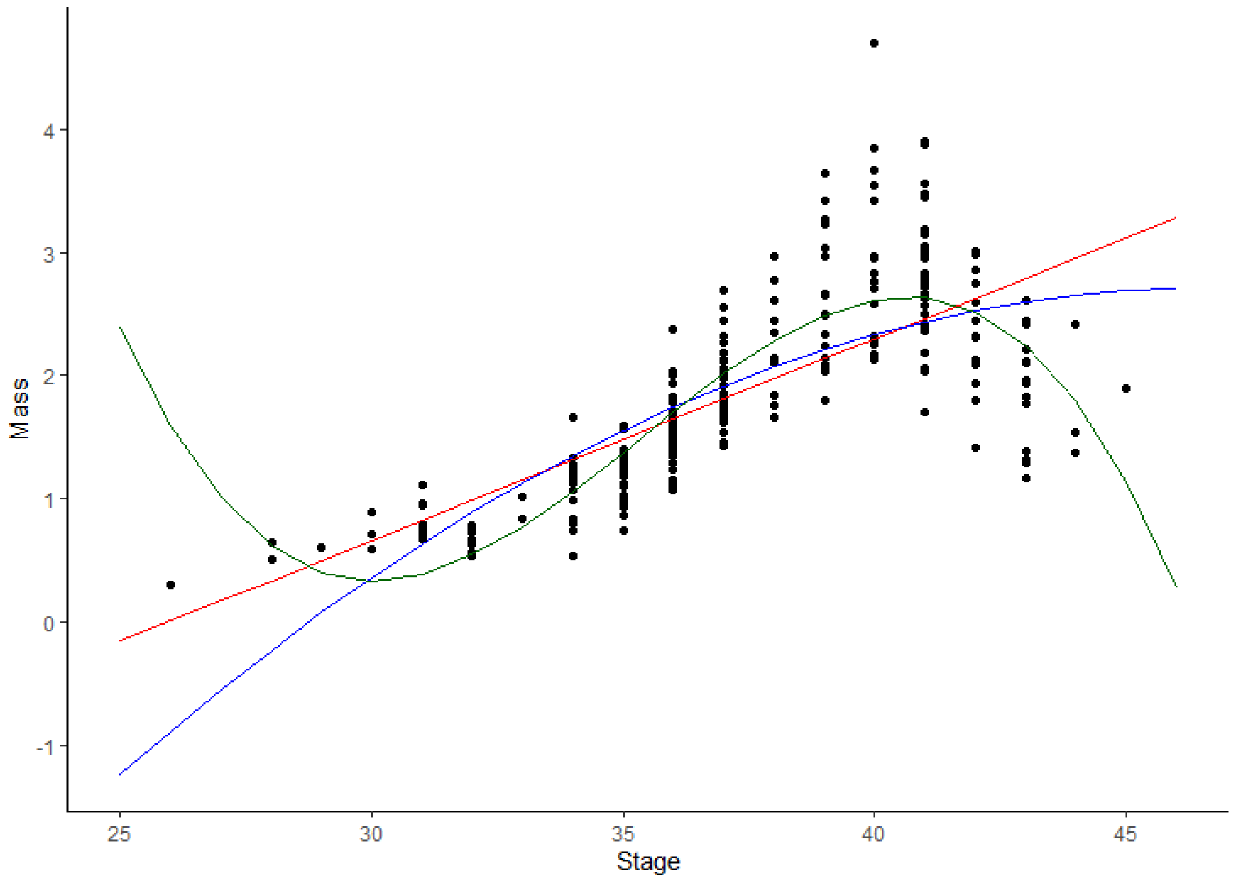

Fig. S4 Scatterplot of *Rana pipiens* tadpole mass (g) by Gosner stage of development following chronic exposure to PFBA. Red line shows first order polynomial relationship. Blue line indicates second order polynomial relationship (Stage^2). Green line indicates a third order polynomial relationship (Stage^3).

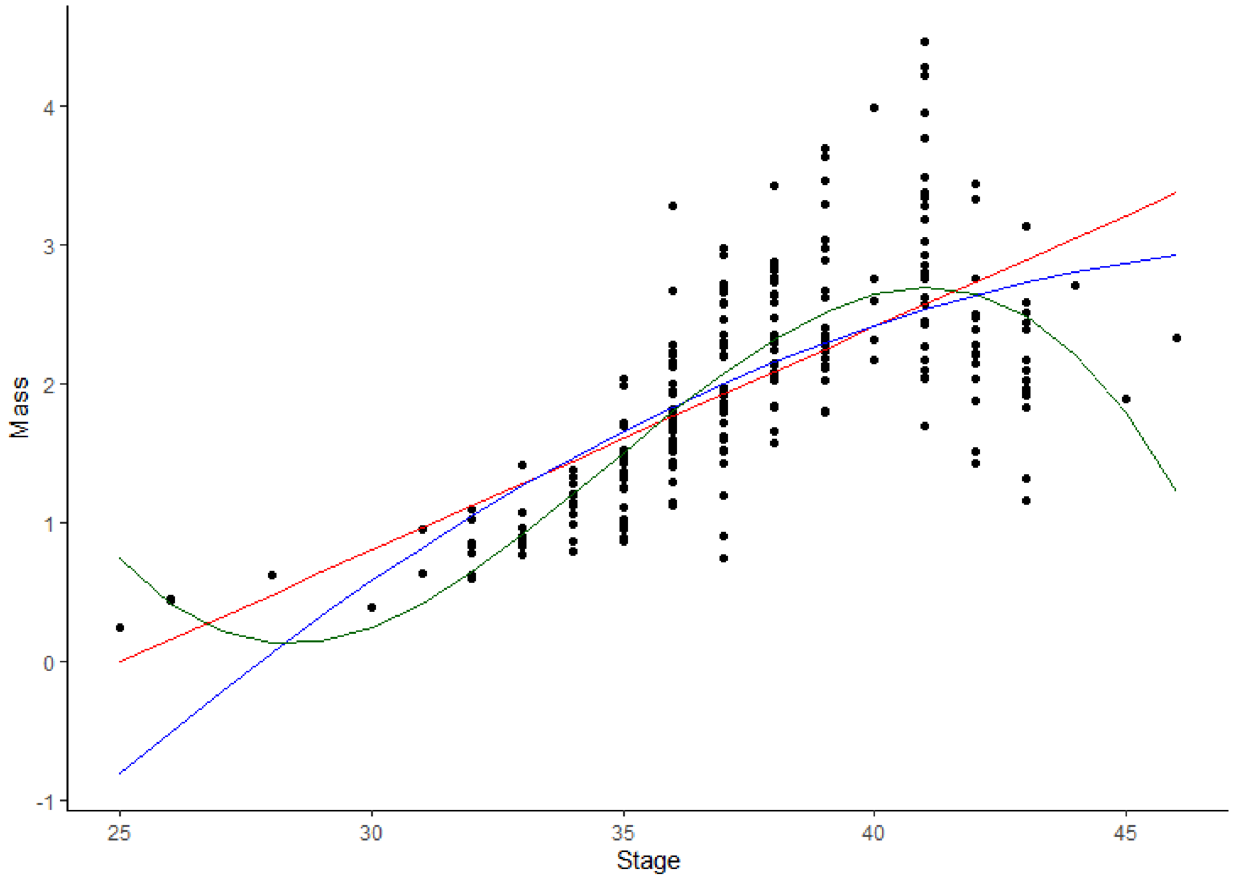

Fig. S5 Scatterplot of *Rana pipiens* tadpole mass (g) by Gosner stage of development following chronic exposure to PFHxA. Red line shows first order polynomial relationship. Blue line indicates second order polynomial relationship (Stage^2). Green line indicates a third order polynomial relationship (Stage^3).

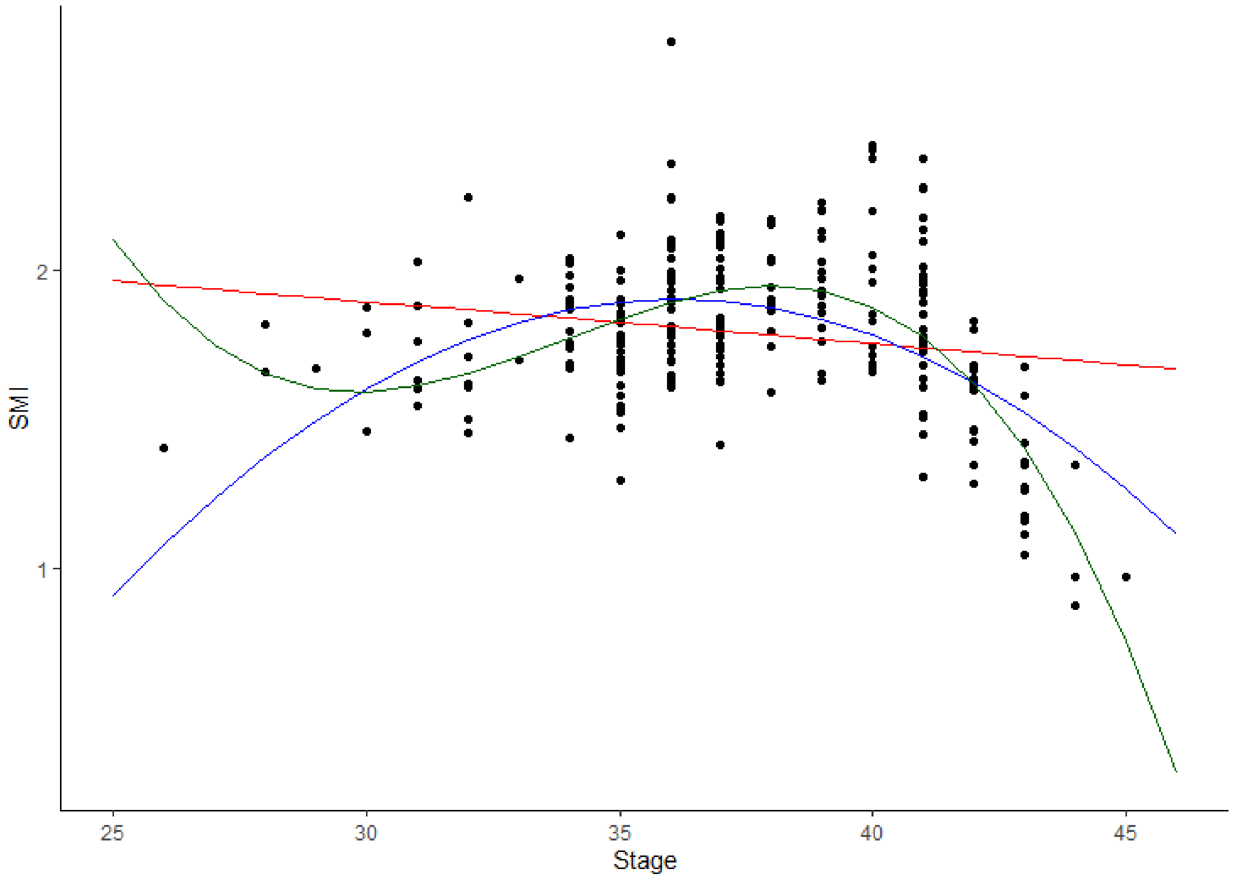

Fig. S6 Scatterplot of *Rana pipiens* tadpole scaled mass index (SMI) by Gosner stage of development following chronic exposure to PFBA. Red line shows first order polynomial relationship. Blue line indicates second order polynomial relationship (Stage^2). Green line indicates a third order polynomial relationship (Stage^3).

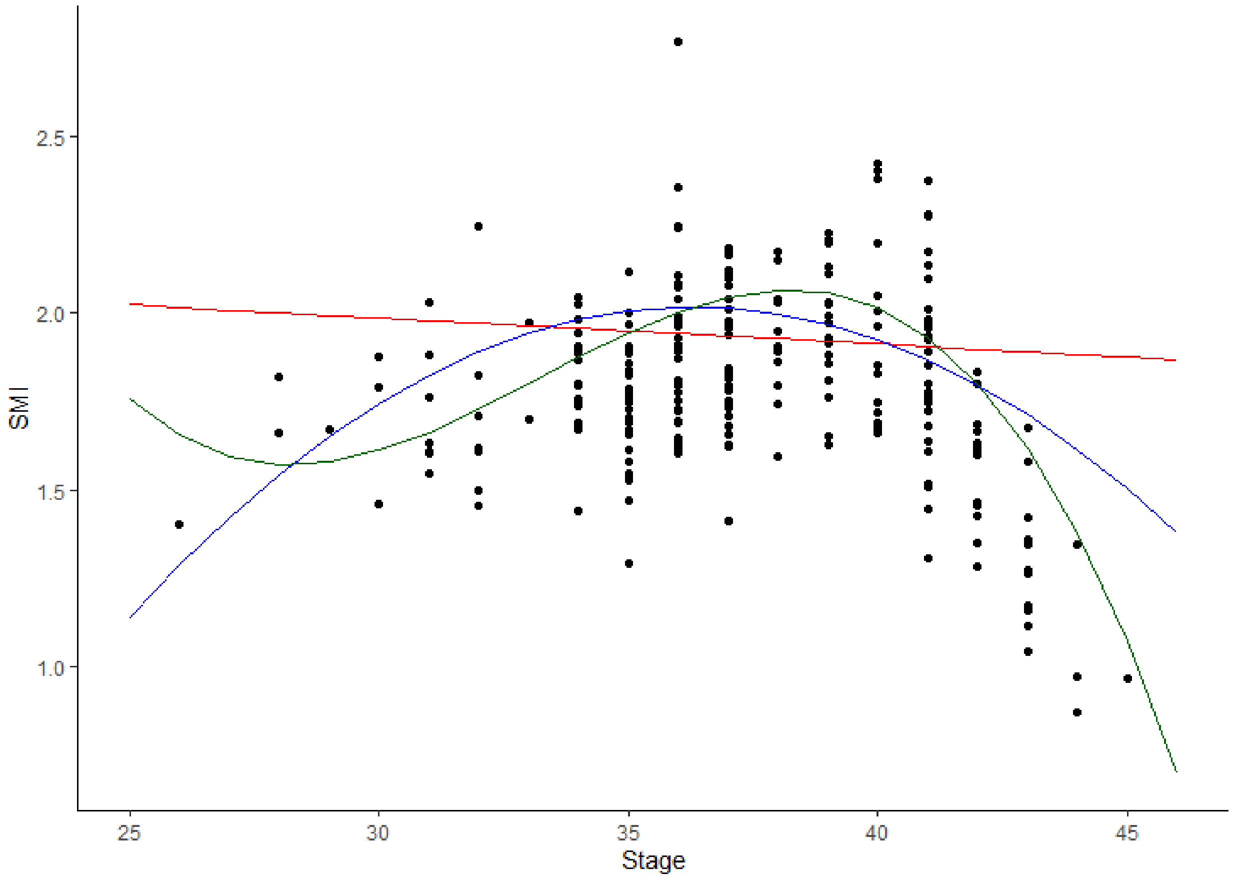

Fig. S7 Scatterplot of *Rana pipiens* tadpole scaled mass index (SMI) by Gosner stage of development following chronic exposure to PFHxA. Red line shows first order polynomial relationship. Blue line indicates second order polynomial relationship (Stage^2). Green line indicates a third order polynomial relationship (Stage^3).

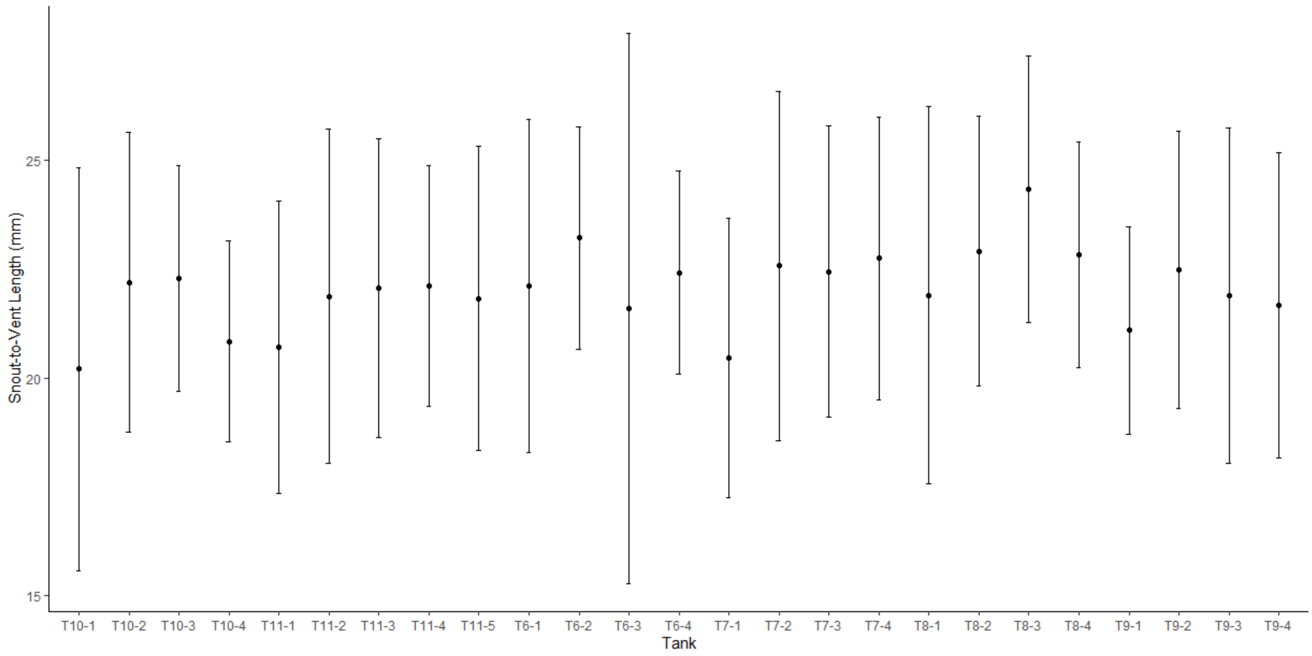

Fig. S8 Scatterplot of mean *Rana pipiens* tadpole snout-to-vent length (SVL) (mm) by treatment tank following chronic exposure to PFHxA highlighting the low variation in tadpole SVL among tanks. Treatment and replicate tank number are shown on the x-axis (i.e., Controls: T11-1 to T11-5, PFHxA 0.1 to 1000 ug/L: T6-1 to T10-4). Error bars correspond to standard deviation.

Table S4. Average water quality parameters (temperature (ºC), dissolved oxygen (% and mg/L), pH, conductivity (µS/cm), ammonia (mg/L), nitrate (mg/L), nitrite (mg/L)), and water hardness (mg/L)), by treatment taken throughout the chronic PFCA exposure study with Rana pipiens tadpoles. Data are reported as mean ± standard deviation.

Treatment	Temp (ºC)	Dissolved Oxygen (%)	Dissolved Oxygen (mg/L)	pH	Conductivity (µS/cm)	Ammonia (mg/L)	Nitrate (mg/L)	Nitrite (mg/L)	Hardness (mg/L)
**Control**	22.2 ± 0.2	86.7 ± 6.2	7.5 ± 0.5	8.3 ± 0.1	184.8 ± 18.9	2.3 ± 2.9	1.4 ± 2.0	0.0 ± 0.0	48.6 ± 13.5
**PFBA_0.1**	22.0 ± 0.2	81.4 ± 7.4	7.1 ± 0.6	8.1 ± 0.1	193.4 ± 18.0	2.3 ± 1.4	2.1 ± 2.2	0.1 ± 0.2	53.7 ± 0.00
**PFBA_1**	22.1 ± 0.3	81.0 ± 10.2	7.0 ± 0.9	8.1 ± 0.2	184.8 ± 22.4	1.8 ± 1.6	2.1 ± 2.2	0.0 ± 0.1	51.1 ± 16.1
**PFBA_10**	21.8 ± 0.3	79.9 ± 4.9	7.0 ± 0.4	8.1 ± 0.1	205.5 ± 11.5	4.3 ± 2.9	4.3 ± 7.2	0.3 ± 0.8	53.7 ± 0.00
**PFBA_100**	22.3 ± 0.2	85.1 ± 6.5	7.4 ± 0.6	8.2 ± 0.2	181.7 ± 30.6	1.6 ± 1.6	0.7 ± 1.2	0.0 ± 0.0	51.1 ± 16.1
**PFBA_1000**	22.1 ± 0.2	78.7 ± 9.5	6.8 ± 0.8	8.1 ± 0.2	199.4 ± 23.8	2.6 ± 2.1	1.8 ± 1.9	0.0 ± 0.1	56.3 ± 12.4
**PFHA_0.1**	22.1 ± 0.2	85.7 ± 10.0	7.4 ± 0.9	8.1 ± 0.2	182.4 ± 19.1	2.3 ± 2.0	1.4 ± 1.3	0.0 ± 0.0	48.6 ± 13.5
**PFHA_1**	22.1 ± 0.2	82.7 ± 9.7	7.2 ± 0.8	8.2 ± 0.3	185.0 ± 27.9	1.5 ± 1.5	0.7 ± 1.2	0.0 ± 0.0	48.6 ± 13.5
**PFHA_10**	22.2 ± 0.2	85.1 ± 9.1	7.4 ± 0.8	8.2 ± 0.3	184.5 ± 36.4	1.8 ± 2.3	0.7 ± 1.2	0.0 ± 0.0	51.1 ± 16.1
**PFHA_100**	22.1 ± 0.2	82.3 ± 9.6	7.2 ± 0.8	8.2 ± 0.3	168.2 ± 33.6	1.4 ± 1.5	1.1 ± 1.3	0.0 ± 0.1	48.6 ± 13.5
**PFHA_1000**	22.0 ± 0.4	79.1 ± 4.4	6.9 ± 0.4	7.9 ± 0.4	196.2 ± 16.6	3.1 ± 1.6	2.5 ± 3.5	0.2 ± 0.6	53.7 ± 0.00

Table S5. Measured concentrations of perfluorobutanoic acid (PFBA) and perfluorohexanoic acid (PFHxA) and percent nominal concentrations detected in treatment solutions throughout the chronic experiment with *Rana pipiens* tadpoles. Analysis conducted on pooled samples by treatment across replicates. Method detection limits are presented when no analytical peak was detected. The method detection limit is the instrumental detection limit multiplied by the dilution factor. Hyphen (-) indicates value not quantified.

PFCA concentration (μg/L)					
**Treatment**	**Sample Collection Time**	**PFBA**	**% Nominal**	**PFHxA**	**% Nominal**
Control[a]	Day 0[b]	0.028	-	< 0.010	-
	Day 42[b]	< 0.012	-	< 0.010	-
PFBA_0.1	Day 0[b]	0.092	92	0.95	-
	Day 3[c]	0.094	94	2.0	-
	Day 24[b]	0.11	110	<0.010	-
	Day 28[c]	0.14	140	0.012	-
	Day 42[b]	0.093	93	< 0.010	-
PFBA_10	Day 0[b]	9.1	91	0.011	-
PFBA_1000	Day 0[b]	930	93	< 1.0	-
	Day 3[c]	935	93.5	< 1.0	-
	Day 24[b]	888	88.8	< 1.0	-
	Day 28[c]	902	90.2	< 1.0	-
	Day 42[b]	845	84.5	< 1.0	-
PFHxA_0.1	Day 0[b]	< 0.012	-	0.11	110
	Day 3[c]	< 0.012	-	0.12	120
	Day 24[b]	< 0.012	-	0.10	100
	Day 28[c]	< 0.012	-	0.074	74
	Day 42[b]	0.025	-	0.096	96
PFHxA_10	Day 0[b]	0.18	-	9.8	98
PFHxA_1000	Day 0[b]	< 1.2	-	957	95.7
	Day 3[c]	< 1.2	-	958	95.8
	Day 24[b]	< 1.2	-	831	83.1
	Day 28[c]	< 1.2	-	875	87.5
	Day 42[b]	< 1.2	-	914	91.4

[a]Dechloraminated water
[b]Water samples collected 1 hour after water change/compound renewal
[c]Water samples collected before water change/compound renewal

Table S6. Results from GLMMs examining differences in log-transformed hepatic-somatic index (HSI) between exposure treatments and the control (intercept) in the chronic exposure study of *Rana pipiens* tadpoles to PFBA and PFHxA. Treatment and Gosner stage of development were included as fixed effects, and tank was included as a random effect. Significant results are in bold.

				**PFBA**					
		**n**	**ß coef**	**SE**	**df**	**t-value**	**p-value**	**Var**	**SD**
**Model 7: logHSI ~ Treatment + Stage + (1|Tank), n = 247**									
Fixed Effects	(Intercept - Control)	50	-1.381	0.101	242.2	-13.737	**<0.001**		
	0.1 µg/L	40	0.021	0.039	23.6	0.538	0.596		
	1 µg/L	38	0.026	0.040	24.5	0.661	0.515		
	10 µg/L	39	0.052	0.040	24.0	1.315	0.201		
	100 µg/L	40	0.064	0.040	23.7	1.617	0.119		
	1000 µg/L	40	0.036	0.039	23.6	0.904	0.375		
	Stage		0.046	0.003	245.6	17.826	**<0.001**		
Random Effects	Tank							0.002	0.040
	Residual							0.019	0.136
				**PFHxA**					
		**n**	**ß coef**	**SE**	**df**	**t-value**	**p-value**	**Var**	**SD**
**Model 8: logHSI ~ Treatment + Stage + (1 | Tank), n = 242**									
Fixed Effects	(Intercept - Control)	50	-1.498	0.110	241.9	-13.645	**<0.001**		
	0.1 µg/L	39	-0.028	0.046	24.6	-0.597	0.556		
	1 µg/L	38	0.001	0.047	24.8	0.027	0.979		
	10 µg/L	38	-0.026	0.047	24.9	-0.550	0.587		
	100 µg/L	38	0.030	0.047	24.8	0.645	0.525		
	1000 µg/L	39	0.055	0.047	24.8	1.191	0.245		
	Stage		0.049	0.003	229.8	17.526	**<0.001**		
Random Effects	Tank							0.003	0.053
	Residual							0.020	0.141

Table S7. Results from logistic regressions examining the probability of tadpoles being male between exposure treatments and the control (intercept) in the chronic exposure study of *Rana pipiens* tadpoles exposed to PFBA and PFHxA. Significant results are in bold.

		**PFBA**		
Predictors	n	Odds Ratio	Confidence Interval	p-value
**Model 9: Sex ~ Treatment, n=174**				
(Intercept-Control)	36	0.38	0.18 – 0.77	**0.010**
0.1 µg/L	28	0.71	0.21 – 2.23	0.562
1 µg/L	27	3.25	1.15 – 9.61	**0.028**
10 µg/L	26	1.91	0.66 – 5.64	0.236
100 µg/L	27	2.41	0.85 – 7.07	0.100
1000 µg/L	30	1.51	0.53 – 4.33	0.441
		**PFHxA**		
Predictors	n	Odds Ratio	Confidence Interval	p-value
**Model 10: Sex ~ Treatment, n=183**				
(Intercept-Control)	36	0.38	0.18 – 0.77	**0.010**
0.1 µg/L	31	1.24	0.43 – 3.57	0.690
1 µg/L	33	1.13	0.40 – 3.23	0.817
10 µg/L	28	1.68	0.59 – 4.90	0.333
100 µg/L	30	0.79	0.25 – 2.40	0.681
1000 µg/L	25	1.73	0.59 – 5.19	0.319

Table S8. Bioconcentration factors (BCF) calculated using average measured exposure concentrations and measured wet weight (w.w.) whole-body and liver concentrations from the 1000 µg/L PFBA and the 1000 µg/L PFHxA treatments in the chronic exposure study of *Rana pipiens* tadpoles. Data are shown for 3 individual tadpoles and resulting average ± SD.

	**PFBA**			**PFHxA**		
Tissue	Tissue concentration (µg/kg w.w.)	Average exposure concentration (µg/L)	BCF (L/kg w.w.)	Tissue concentration (µg/kg w.w.)	Average exposure concentration (µg/L)	BCF (L/kg w.w.)
Whole-Body	1814	900	2.02	7.78	907	0.0086
	2966	900	3.30	10.4	907	0.0114
	1374	900	1.53	23.5	907	0.0259
	**Average ± SD**		**2.28 ± 0.91**			**0.0153 ± 0.0093**
Liver	2493	900	2.77	27.9	907	0.0308
	4327	900	4.81	20.7	907	0.0228
	6629	900	7.37	79.9	907	0.0881
	**Average ± SD**		**4.98 ± 2.30**			**0.0472 ± 0.0356**
